# The Impact of Psychological Factors on Periodontitis in the Elderly: A Systematic Review

**DOI:** 10.1055/s-0045-1809438

**Published:** 2025-07-07

**Authors:** Reghunathan S. Preethanath, Wael I. Ibraheem, Abdullah A. Meshni, Tazeen Dawood, Mohammad Nazish Alam, Sukumaran Anil

**Affiliations:** 1 Department of Preventive Dental Sciences, College of Dentistry, Jazan University, Jazan, Saudi Arabia; 2 Department of Prosthetic Dental Sciences, College of Dentistry, Jazan University, Jazan, Saudi Arabia; 3 Department of Dentistry, Oral Health Institute, Hamad Medical Corporation, Doha, Qatar; 4 College of Dental Medicine, Qatar University, Doha, Qatar

**Keywords:** periodontitis, psychological factors, older adults, GRADE analysis, oral health, stress, anxiety, depression, cognitive impairment, cardiovascular outcomes

## Abstract

Psychological factors and periodontitis are prevalent in geriatric populations, with evolving evidence suggesting potential associations. This systematic review comprehensively examines and evaluates the bidirectional relationship between psychological factors and periodontitis in older adults. Following PRISMA guidelines, systematic searches were conducted across PubMed, Google Scholar, Scopus, Embase, and Web of Science databases. The research question was structured using the PECO framework (P = older adults aged ≥ 60 years; E = presence of psychological factors; C = absence of psychological factors; O = periodontitis and its severity). Articles were selected based on predetermined inclusion/exclusion criteria, followed by data extraction. The Newcastle-Ottawa Scale assessed risk of bias and methodological quality, while the Grading of Recommendations, Assessment, Development, and Evaluations (GRADE) framework determined evidence certainty. Of 475 studies identified, 13 met the inclusion criteria for qualitative synthesis (8 on depression, 3 on stress, 1 on cognitive impairment, and 1 on examining multiple factors). For depression–periodontitis associations, results were inconsistent: four studies demonstrated positive associations, three found no significant relationship, and one had unclear findings. All three studies investigating stress–periodontitis relationships showed positive associations, suggesting a more consistent connection. The single study on cognitive impairment found significant independent associations with periodontitis after controlling for confounders. Only one longitudinal study ( *n* = 11,454) revealed increased periodontitis risk at both 6-month and 1-year follow-ups in older adults with depression. Based on Newcastle-Ottawa Scale ratings, seven studies were deemed high quality, three moderate quality, and one low quality. GRADE assessment indicated very low certainty of evidence across all psychological factors, primarily due to methodological heterogeneity. This systematic review indicates significant associations between periodontal disease severity and psychological factors, particularly stress and cognitive impairment, in older adults. These findings suggest the importance of integrating psychological assessment into geriatric oral healthcare. More longitudinal research is needed to establish causality and bidirectional relationships, especially in middle- and low-income countries with a rapidly growing geriatric population. Mental health considerations should be incorporated into prevention and treatment strategies for periodontal disease in older adults.

## Introduction

With declining fertility rates and rising life expectancies, the global elderly population (60 years and older) has grown substantially from 5% in 1975 to 11.5% in 2019 and is projected to reach 2.1 billion by 2050. 1 2 Although developed nations currently have a higher percentage of older adults, rapid growth occurs in developing countries. 3 The population of persons aged 80+ years is increasing by 4.0% annually. 3 Reflecting this demographic shift, more older adults are retaining natural teeth. In the United States, edentulism decreased from 38% in 1980 to 4.6% in 2012. 4 Similar patterns are seen in other developed nations like Europe, Japan, and Australia, with declining edentulism rates among older adults. 5 6 7 With more retained teeth, periodontitis prevalence may rise in older populations.

Periodontitis is an inflammatory disorder affecting the soft tissue and bone supporting the tooth. 8 It has a complex pathogenesis initiated by subgingival colonization of gram-negative anaerobic bacteria. Interactions between the microbial challenge and host inflammatory response determine disease progression and connective tissue/bone destruction. Periodontitis has a multifactorial etiology with varied clinical presentations. A new classification grades periodontitis (A, B, C) by disease progression risk, treatment response, and potential oral health impact, with grade C being the most rapidly progressive. 9 It stages disease (I, II, III, IV) by severity and complexity, with stage IV being the most severe with altered masticatory function. Stages are designated localized, generalized, or molar-incisor patterns based on affected teeth. Various clinical measurements define periodontal disease cases in research, including probing depth, gingival bleeding, and clinical attachment level. Probing depth in millimeters (mm) from the free gingival margin to the base of the pocket is commonly used. 10 Gingival bleeding indicates active inflammation. Clinical attachment level, measured from the cementoenamel junction to the base of the pocket, is used in epidemiology. Some studies measure alveolar bone loss radiographically to quantify destruction. 8 Differing measures and thresholds to define disease across studies of older adults may hamper prevalence comparison. Partial versus complete mouth assessments also underestimate true prevalence and severity. 11

Numerous studies have documented periodontitis prevalence and severity in older adults, but progression data are limited, possibly due to the low proportion of adults aged 65+ retaining natural teeth. However, recent NHANES survey data (2018–2019) indicate 63% of older adults have moderate-severe periodontitis, with a small but significant proportion having severe disease. 12 The New England Elders Dental Study of 554 adults aged 70+ similarly found that 66% had moderate periodontitis, with at least one 4- to 5-mm pocket. 7 13 Other U.S. 14 and global studies 9 15 16 report comparable results. Severe 6–7 mm pockets were seen only in one-fifth of older adults. Likewise, only a small percentage have advanced alveolar bone loss. 17 18 While periodontitis prevalence and severity rise, current consensus disputes age as an independent risk factor. Rather than increased vulnerability, the cumulative effect of prolonged exposure to established periodontal disease risk factors is cited. 19

Systemic illness is common in older adults. Age-related declines in health, cognition, financial capacity, physical ability, and other functions are expected. Older adults often experience stress due to physical frailty and limited activity. Stressed elderly are more susceptible to periodontitis, as poor oral hygiene, increased glucocorticoid release, *suppressed* immunity, and insulin resistance are all linked to stress and may increase periodontitis risk. 20 Periodontitis is also associated with other chronic diseases like cardiovascular disease, rheumatoid arthritis, atherosclerosis, and diabetes, reinforcing the role of periodontitis-induced inflammatory mediators entering the bloodstream. 21

Recent evidence has significantly strengthened our understanding of the relationship between periodontitis and systemic diseases, as comprehensive research by Sanz et al 22 has demonstrated that periodontal inflammation directly contributes to cardiovascular pathology through bacteremia and inflammatory pathways. Their work emphasizes how periodontal pathogens and inflammatory mediators can affect distant organs beyond the oral cavity. Additionally, Ramseier et al 23 have established that effective periodontal treatment can significantly improve cardiovascular disease outcomes, highlighting the clinical importance of maintaining periodontal health in at-risk populations such as the elderly. These findings underscore the need to identify factors influencing periodontal disease progression in geriatric patients.

Given the role of periodontitis in systemic and neuroinflammation, 20 an association between periodontitis and psychological impairment is biologically plausible and clinically relevant. The complex bidirectional relationship between oral health and mental health represents a critical yet understudied aspect of geriatric healthcare. Psychological factors may influence periodontitis development through behavioral mechanisms (affecting oral hygiene practices), neuroendocrine pathways (stress-induced immunomodulation), and inflammatory processes (shared inflammatory mediators). Conversely, periodontitis may exacerbate psychological conditions through chronic inflammation and its effects on neurological function.

Understanding these relationships has significant implications for developing integrated approaches to geriatric healthcare. Clarifying associations between psychological factors and periodontitis could help identify at-risk elderly populations and inform more effective preventive and therapeutic strategies. Thus, this systematic review aims to comprehensively assess and evaluate the relationship between psychological factors and periodontitis in older adults, addressing an important gap in our understanding of geriatric oral health determinants.

## Methods

This systematic review followed PRISMA guidelines and the Cochrane Handbook. 24 Before initiation, the protocol was registered on PROSPERO (Reg. no: CRD42023424929).

### Focused Question

Is there an association between psychological factors and periodontitis in older adults? The PECOS framework was used: P = older adults aged ≥60 years; E = presence of psychological factors; C = older adults without psychological factors; O = periodontitis severity. This structure ensures consistent alignment between our research question formulation and methodology.

### Search Strategy

Two reviewers independently searched PubMed, Cochrane CENTRAL, Embase, Scopus, and other sources like Google Scholar and reference lists, without language restrictions. Ongoing trial registries were searched. Authors were contacted regarding unpublished studies. Manual searches of periodontology and psychology journals were conducted. Reference lists of excluded articles were checked. The detailed search strategy is presented in Table 1 .

**Table 1:** Search strategy on PubMed

Sl. no.	Domains	Keywords	Wildcard
1	Periodontitis	(Periodontal Loss, Periodontal Attachment) OR (Alveolar Process a ) OR (Alveolar Resorption a ) OR (Resorption a , Alveolar) OR (Bone Loss a , Periodontal) OR (Periodontal Bone Loss a ) OR (Periodontal Resorption) OR (Resorption, Periodontal) OR (Alveolar Bone Atroph a ) OR (Bone Atroph a , Alveolar) OR (Bone Loss a , Alveolar) OR (Intra-bony Defects) OR (Bony defect a ) OR (Defect a , bony) OR (Bone defect a ) OR (Defect a , bone)	atrophy, atrophies, resorption, resorptions, loss, losses, bone, bony, defect, defects
2	Psychological factors	(“Depression” OR “depressive disorders” OR “depression symptoms”) OR (“anxiety” OR “mood disorders” OR “psychological distress”) OR (“stress” OR “psychological distress” OR “stress symptom”) OR (“cognitive impairment” OR “cognitive decline” OR “cognitive dysfunction”)	None
3	Population	“older adults” OR “elderly” OR “geriatric population” OR “older people” OR “senior citizens” OR “aged 60 and above” OR “vulnerable population”	None
4	Assessment Tools	(“Periodontal Probing index” OR “Community Periodontal Index for Treatment Needs” OR “Periodontal Screening and Recording Index”) AND (“HADS” OR “hospital Anxiety and Depression Index” OR “Becks depression Index” OR “BDI” OR “LIPPS Inventory for stress in elderly” OR “DASS” OR “Depression anxiety and stress scale” OR “Mini-Mental State Examination” OR “MMSE” OR “Geriatric Depression Scale” OR “GDS”)	None
5	Outcome	(Outcome, Treatment) OR (Patient-Relevant Outcome) OR (Outcome a , Patient-Relevant) OR (Patient Relevant Outcome) OR (Treatment needs for elderly) OR (Oral health-related quality of life)	outcome, outcomes

a Indicates wildcard in PubMed.

For the literature search, we employed comprehensive search terms combining multiple domains: (1) periodontitis and related periodontal conditions; (2) psychological factors including depression, anxiety, stress, and cognitive impairment; (3) geriatric population descriptors; (4) relevant assessment indices; and (5) outcome measurements. The search was structured to capture all relevant publications through October 2024, with no language restrictions applied.

### Eligibility Criteria

Original cross-sectional, case–control, longitudinal, and observational studies investigating associations between periodontitis and psychological factors (depression, stress, anxiety, cognitive impairment) in adults aged ≥60 years were included. Studies had to report sample size calculations and use validated tools to assess periodontal health/disease and psychological factors. Self-reported and clinically diagnosed periodontitis were eligible, with a preference for clinical diagnosis. Severe periodontitis was selected over moderate or mild forms. Reviews, letters, in vitro studies, animal studies, case reports, and conference abstracts were excluded. Studies on other oral conditions were not included.

Studies were selected based on clearly defined inclusion criteria regarding population (older adults ≥60 years), exposures (validated measures of psychological factors), comparators (appropriate control or reference groups), outcomes (clinically or radiographically assessed periodontitis), and study design (original research with proper methodology). This approach ensured systematic and reproducible study selection aligned with our research question.

### Screening and Selection

Search results were imported into EndNote and Excel. Titles/abstracts were independently screened by two reviewers (R.S.P., A.A.) for relevance. Non-research articles were excluded. Full texts were obtained for potentially relevant articles. Two reviewers (R.S.P., A.A.M.) then assessed full texts for eligibility. Reference lists of included studies were searched. Disagreements were resolved through discussion or a third reviewer (S.A.). The screening process followed a two-stage approach to ensure comprehensive and unbiased selection. Initial screening focused on title and abstract evaluation against predetermined criteria, followed by full-text review of potentially eligible studies. We specifically sought articles examining associations between validated measures of psychological factors and periodontitis in adults ≥60 years. Two reviewers conducted each step independently, with a third reviewer resolving discrepancies.

### Data Extraction

Two reviewers (R.S.P., W.I.I.) independently extracted data using a standardized form recording: study design, sample demographics, psychological factor(s) studied, diagnostic/rating tools used, analyses, and key findings. One reviewer (A.A.M.) completed the extraction, and two (R.S.P., S.A.) verified it for accuracy. The data extraction process employed structured forms designed for this review, ensuring comprehensive and consistent information capture across all included studies. Particular attention was given to extracting details about psychological assessment methodologies, periodontal examination protocols, demographic characteristics of participants, and statistical analyses employed, facilitating robust comparison across studies.

### Risk of Bias Assessment

The Newcastle-Ottawa Scale assessed the methodological quality of included studies. 25 Using NOS guidelines, each study was assigned a score from 0 to 10 stars. Two reviewers (R.S.P., S.A.) evaluated three domains: sample selection, exposure assessment, and comparability. Scores ≥8 indicated low risk of bias. Scores <4 on ≥2 domains indicated high risk. For cross-sectional studies, we evaluated four selection criteria (representativeness, sample size, nonrespondents, and exposure ascertainment), comparability criteria (control for confounding), and outcome criteria (assessment methods and statistical analysis). For case–control studies, selection criteria included case definition, representativeness, control selection, and control definition; exposure criteria covered exposure ascertainment, assessment methods, and nonresponse rates. This comprehensive approach systematically evaluated internal validity for each included study.

### Quantitative Analysis

Due to inconsistencies in participants' age, periodontal assessments, and psychological factor measurements, conducting a meta-analysis was not feasible. Significant heterogeneity was expected. Thus, findings were summarized using the GRADE system.

We thoroughly evaluated the potential for meta-analysis, examining statistical heterogeneity (using *I* ^2^ statistics), methodological heterogeneity (differences in study design and quality), and clinical heterogeneity (variations in participant characteristics, interventions, and outcome measurements). Despite identifying several studies with similar psychological constructs and periodontal outcomes, the pronounced methodological differences in assessment tools and reporting formats precluded meaningful statistical pooling. We therefore employed narrative synthesis with GRADE assessment to systematically evaluate evidence quality.

### Level of Evidence

The GRADE approach examined the level of evidence. GRADE rates the quality of evidence and strength of recommendations. The level of evidence was rated as low or very low if studies had serious limitations in risk of bias, inconsistency, imprecision, indirectness, or publication bias. GRADE does not assess intervention effects/dosages. Here, it evaluated the evidence for psychological factor–periodontitis associations in older adults. Our GRADE assessment specifically evaluated five domains that might reduce confidence in estimated effects: risk of bias (methodological limitations), inconsistency (unexplained heterogeneity), indirectness (population, intervention, outcome differences from review question), imprecision (wide confidence intervals, small sample sizes), and publication bias (systematic underestimation of effects). Each domain was rated as not serious, serious, or very serious, with the final evidence quality classified as high, moderate, low, or very low. This structured approach provided a transparent evaluation of evidence certainty for each psychological factor–periodontitis relationship.

## Results

**Fig. 1:**
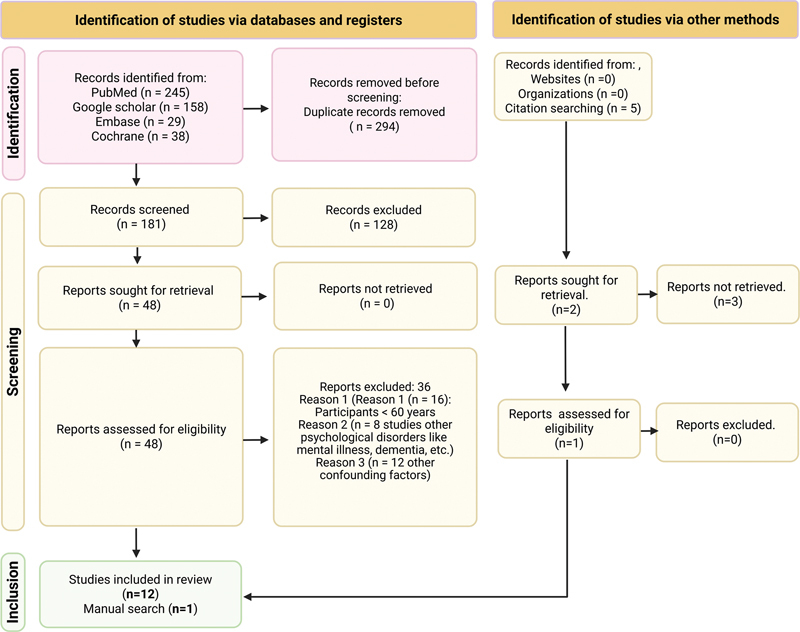
PRISMA flowchart.

The database search yielded 475 potential articles, of which 294 were duplicates. After title/abstract screening of the remaining 181 articles, 128 were excluded. The 48 articles receiving full-text review were reduced to 13 included studies after applying eligibility criteria ( Fig. 1 ). The remaining were removed for a reason ( Supplementary Tables S1 and S2 [available in the online version only]). All studies underwent GRADE evidence assessment. The main characteristics of the 13 included studies are presented in Table 2 , categorized by exposure–outcome relationship direction. Four studies examined depression–periodontitis links, three investigated stress–periodontitis associations, two assessed cognitive impairment–periodontitis relationships, and four found no association between psychological factors and periodontitis. Results are presented by psychological factors below.

**Table 2:** Characteristics of included studies

Authors/Country	Study design	Sample size (M/F)	Age group (years)	Psychological factors and periodontitis assessment	Key findings
Viana et al 26 Brazil	Cross-sectional	191 (35/156)	60–85	Depression: GDS-15 periodontitis: clinical examination	No association between periodontitis and depression; an association between oral hygiene and depression was observed
Shin et al 37 South Korea	Cross-sectional	189 (98/91)	60–90	Cognitive impairment: MMSE-KC depression: CES-D periodontitis: clinical examination and radiographs	Periodontitis is independently associated with both cognitive impairment and depression after controlling for confounders
Botelho et al 34 Portugal	Cross-sectional	592 (272/320)	≥ 65	Stress: PSS-10-PT Oral HRQoL: OHIP-14-PT periodontitis: clinical examination	Self-perceived xerostomia and stress are significantly associated with periodontitis severity
Persson et al 29 USA	Cross-sectional	701 (287/414)	60–75	Depression: GDS periodontitis: clinical examination	No significant association between depression and periodontitis in elderly participants
Luca et al 32 Italy	Case–control	90 (31/59)	≥ 60	Depression: DSM-IV criteria, periodontitis: clinical examination	Significant correlation between psychiatric variables and poor periodontal health status
Skośkiewicz-Malinowska et al 30 Poland	Cross-sectional	500 (180/320)	65–99	Depression: PHQ-9 periodontitis: clinical examination	No association between periodontitis and depression; an association between oral hygiene and depression was detected
Coelho et al 36 Brazil	Cross-sectional	621 (321/300)	60–85	Stress: Perceived Stress Scale periodontitis: clinical attachment loss	A significant association between stress and periodontitis is attributed to the multifactorial nature of both conditions
Hugo et al 38 Brazil	Cross-sectional	230 (not specified)	60–75	Stress: ISSL depression: BDI periodontitis: GBI and probing depth	A complex relationship between stress and periodontitis was identified; no association between depression and periodontitis
Hilgert et al 35 Brazil	Cross-sectional	235 (not specified)	60–84	Stress: ISSL periodontitis: clinical examination cortisol: salivary assay	No association found between cortisol levels, stress measures, and periodontitis parameters
Youn et al 31 Korea	Cross-sectional	2002 (872/1134)	≥ 65	Depression: ISSL sleep: questionnaire periodontitis: clinical examination	A significant association was found between depression, sleep disturbance, and periodontitis severity
Kim et al 28 Korea	Longitudinal	11,454 (not specified)	≥ 66	Depression: ISSL periodontitis: clinical diagnosis (database)	Significant association between depression and chronic periodontitis at 6-mo and 1-y follow-ups
Iwasaki et al 27 Japan	Cross-sectional	85 (not specified)	≥ 75	Depression: not specified, cognitive decline: assessment, periodontitis: CAL ≥6 mm	A significant association was found between cognitive decline and severe periodontitis
Kjellström et al 33 Sweden	Case–control	1,610 (not specified)	65–85	Depression: assessment periodontitis: panoramic radiography	No significant difference in depression symptoms between those with and without periodontitis

Abbreviations: OHIP-14-PT, Oral Health Impact Profile-14 Portuguese version; PSS-10-PT, Perceived Stress Scale-10 Portuguese version; DSM-IV, Diagnostic and Statistical Manual of Mental Disorders, 4th edition; PHQ-9, Patient Health Questionnaire-9; ISSL, Lipp's Inventory of Stress Symptoms for Adults; BDI, Beck's Depression Index; GBI, Gingival Bleeding Index; CAL, Clinical Attachment Loss; HRQoL, health-related quality of life.

### Depression–Periodontitis

Eight studies examined depression–periodontitis associations, considering depression as the exposure and periodontitis as the outcome. Studies were conducted between 2003 and 2022 with sample sizes ranging from 191 to 11,545. One study was from Brazil 26 ; the rest were from high-income countries. There were four cross-sectional, 27 28 29 30 31 32 33 three case–control, 27 32 33 and one longitudinal study. 28 Various depression scales were used. Periodontitis was assessed by clinical examination (six studies) or radiographs (two studies). In the longitudinal study by Kim et al 28 in 11,545 older South Koreans, depressive mood was associated with periodontitis at 6-month and 1-year follow-ups. All studies were controlled for confounders like age, sex, and smoking. Meta-analysis was precluded by heterogeneity across studies. Of the eight studies examining depression–periodontitis relationships, four demonstrated positive associations, while three found no significant relationship, and one showed unclear associations. This inconsistency in findings may reflect methodological differences in depression assessment tools, with studies employing various instruments including the Geriatric Depression Scale (GDS), Beck's Depression Inventory (BDI), and Patient Health Questionnaire (PHQ-9). Additionally, different approaches to periodontitis assessment (clinical vs. radiographic) may have contributed to the variability in results.

### Periodontitis and Stress

Three cross-sectional studies examined the association between stress (exposure) and periodontitis (outcome). Sample sizes ranged from 235 to 621. One study was conducted in Portugal 34 and two in Brazil. 35 36 Different validated stress scales were used, including the Perceived Stress Scale (two studies) and Lipp's Inventory of Stress Symptoms for Adults (one study). Periodontitis was assessed via clinical examination for pocket depth ≥4 mm (one study), clinical attachment loss >4 mm (one study), Community Periodontal Index (one study), and radiographic bone loss >0 mm (one study). Two studies had a moderate risk of bias 34 36 and one had a low risk. 35 Confounding was controlled statistically. The small number and heterogeneity between studies precluded meta-analysis. All three studies examining stress–periodontitis relationships demonstrated positive associations, suggesting a more consistent relationship than observed with depression. This consistency is particularly noteworthy given the methodological variations across studies. The findings align with established biological mechanisms linking stress to periodontal health through immune function alterations, behavioral changes affecting oral hygiene, and potential direct effects on periodontal tissues.

## Periodontitis and Cognitive Impairment

One study examined associations between periodontitis, cognitive impairment, and depression. 37 This cross-sectional study was conducted in South Korea in 2016 with 189 older adults. Periodontitis was assessed by clinical examination of pocket depth and clinical attachment loss using digital panoramic radiographs. Cognitive impairment was measured with the Mini-Mental State Examination Korean version (MMSE-KC) and depression with Beck's depressive scale. This study was rated as having good methodological quality. Statistical analysis revealed that cognitive impairment and depression were independently associated with chronic periodontitis, after controlling for confounders like age, sex, occupation, and smoking ( Table 2 ).

It is important to note that our systematic search identified numerous studies investigating the relationship between periodontitis and cognitive impairment. Still, many did not meet our strict inclusion criteria regarding age group (≥60 years exclusively), assessment tools, or reporting methods. The literature in this field has expanded substantially in recent years, with a notable meta-analysis by Guo et al 53 incorporating 20 studies on this topic. Their analysis demonstrated significant associations between periodontal disease and cognitive impairment across diverse populations. The neuroinflammatory pathway linking periodontitis and cognitive decline has received increasing research attention. Periodontal pathogens and their byproducts may contribute to neuroinflammation through systemic circulation, potentially exacerbating neurodegenerative processes. This mechanism aligns with findings from Wu et al 54 and Dziedzic A 55 , who identified significant relationships between periodontal parameters and cognitive function in older adults. While our review identified only one study meeting all inclusion criteria, the broader literature strongly suggests this remains an important area for further investigation, with a specific focus on geriatric populations.

## Periodontitis and Depression, Stress

One cross-sectional study 38 conducted in Brazil, with 230 older adults evaluated, examined the associations between chronic periodontitis (outcome) and stress and depression (exposures). Periodontitis was assessed using the gingival bleeding index and probing depth. Stress was measured with the Lipp's Inventory of Stress Symptoms for Adults (ISSL) and depression with Beck's Depression Index (BDI). Results showed a complex relationship between stress and periodontitis, but no association between depression and periodontitis. This study by Hugo et al. 38 provides valuable insights into the complex interplay between multiple psychological factors and periodontitis. Their findings suggest that the relationship between stress and periodontitis may involve various pathways, not limited to immune system changes alone. The differential findings for stress versus depression highlight the importance of examining specific psychological constructs rather than general “psychological factors” when investigating relationships with periodontal health.

## Methodological Quality

Methodological quality was assessed using the Newcastle-Ottawa Scale (NOS) for cross-sectional and case–control studies ( Tables 3 and 4 ). Based on NOS ratings, seven studies were deemed high quality, three moderate quality, and one low quality with high risk of bias. The main limitations in lower-quality studies were a lack of case verification, inadequate validation of exposures, and omission of nonresponse rates. 33 Moderate issues with case selection, sample size, and response rates existed in three studies. 29 34 36 Most studies 26 27 28 31 32 35 38 had a low risk of bias for exposure–outcome assessment using valid instruments. Questionnaire-based studies appeared more susceptible to bias. Despite similar settings and cohorts, meta-analysis was precluded by differences in periodontitis, psychological factors, outcome measurements, and varying methodological quality. This would introduce substantial heterogeneity and inconsistency. Thus, findings are summarized using the GRADE approach.

**Table 3:** Risk of bias for cross-sectional studies by Newcastle-Ottawa tool 25

Assessment domain	Evaluation criteria	Viana et al 26	Shin et al 37	Botelho et al 34	Persson et al 29	Youn et al 31	Coelho et al 36	Hugo et al 38	Hilgert et al 35
**Selection**	Representativeness of sample	*	**	**	**	*	**	**	**
	Sample size	**	*	*	**	**	*	*	*
	Nonrespondent	*	*	*	–	–	*	*	*
	Uncertainty of exposure	–	–	–	–	*	–	–	–
**Comparability**	Control for confounding	N/A	*	N/A	N/A	*	N/A	N/A	N/A
**Outcome**	Assessment of outcome	*	*	*	*	*	*	*	*
	Statistical tests	**	**	*	*	*	*	*	*
**Overall quality rating**		**Good**	**Good**	**Moderate**	**Moderate**	**Good**	**Moderate**	**Good**	**Good**

Notes:

• Rating system: * = one star, ** = two stars, - = zero stars, N/A = not applicable.

• Overall quality rating: good = 7–10 stars; moderate = 5–6 stars.

• The Newcastle-Ottawa Scale evaluates methodological quality across three domains: selection of study groups, comparability of groups, and assessment of outcomes.

**Table 4:** Risk of bias for case–control studies by the Newcastle-Ottawa tool, 25

Assessment domain	Evaluation criteria	Luca et al 32	Iwasaki et al 27	Kjellström et al 33
**Selection**	Case definition adequate	*	*	**
	Representativeness of cases	**	**	*
	Selection of controls	*	*	–
	Definition of controls	*		–
**Comparability**	Control for confounding	**	N/A	N/A
**Exposure**	Ascertainment of exposure	–	*	–
	Same method of ascertainment for case and control	*	*	*
	Nonresponse rate	–	–	–
**Overall quality rating**		**Good**	**Good**	**Poor**

Notes:

• Rating system: * = one star, ** = two stars, - = zero stars, N/A = not applicable.

• Overall quality rating: good = 7–10 stars; poor = 0–4 stars.

• The Newcastle-Ottawa Scale evaluates methodological quality across three domains: selection of study groups, comparability of groups, and assessment of exposure.

## GRADE Tool for Evidence

The GRADE approach evaluated the level of evidence for associations between periodontitis and depression, stress, and cognitive impairment in older adults. Overall, the evidence was rated as low or very low quality in most studies. Several limitations may have impacted study outcomes, including flaws in methodology, design, sample size, and validation of measures. 39 40 Heterogeneity in these factors likely contributed to inconsistent results across studies ( Table 5 ). Our GRADE assessment revealed that the evidence quality was constrained primarily by methodological limitations, inconsistency across studies, and indirectness in addressing the primary research question. For depression–periodontitis associations, the evidence was particularly heterogeneous, with some studies showing positive associations and others finding no relationship. The proof of stress–periodontitis associations was more consistent but still limited by methodological concerns. The limited number of studies meeting inclusion criteria for cognitive impairment restricted our ability to establish high-quality evidence, despite promising findings from the included research and broader literature. The overall low to very low GRADE ratings do not necessarily indicate an absence of relationships between psychological factors and periodontitis, but rather highlight the need for more methodologically rigorous studies specifically focused on older adult populations. Future research should employ standardized assessment tools, clearly defined periodontal parameters, and longitudinal designs to strengthen the evidence base in this important area.

**Table 5:** Level of certainty

No. of studies	Study design	Risk of bias	Inconsistency	Indirectness	Imprecision	Publication bias	Impact	Certainty
**Periodontitis and depression (** ***n*** ** = 8)**	Cross-sectional a ( *n* = 4) Case–control a ( *n* = 3) Longitudinal a ( *n* = 1)	Very serious b	Very serious c	Very serious c	Not serious	Highly suspected d	Of the 8 depression-periodontitis studies, 4 found a positive association, 3 found no association, and 1 was inconclusive	⨁◯◯◯ Very low
**Periodontitis and stress (** ***n*** ** = 3)**	Cross-sectional ( *n* = 3)	Very serious b	Very serious c	Very serious c	Not serious	Highly suspected d	All 3 studies found a positive association between stress and periodontitis in the elderly	⨁◯◯◯ Very low
**Periodontitis and cognitive impairment (** ***n*** ** = 1)**	Cross-sectional a ( *n* = 1)	Very serious b	Very serious c	Very serious c	Not serious	Highly suspected d	Cognitive impairment and depression were independently associated with periodontitis	⨁◯◯◯ Very low

Abbreviation: CI, confidence interval.

a Observational studies are at higher risk of bias due to the criteria of exposed and unexposed population.

b Heterogeneity of instruments to measure periodontitis and depression, stress, and cognitive impairment.

c The majority of evidence is from the geriatric population of either home care, small sample from selected population not from geriatric population in general.

d Most of the observational studies are prone to publication bias as they do not have registration procedures like randomized control and clinical trials.

## Discussion

This systematic review used GRADE analysis to examine associations between psychological factors and periodontitis progression in older adults. Our findings revealed important patterns across the included studies: positive associations were found when stress and cognitive impairment were investigated as exposures for periodontitis in five included studies. The stress–periodontitis relationship demonstrated consistent results across all three studies examining this factor. However, results were inconsistent across the nine studies exploring depression–periodontitis links, with most showing no clear association. This aligns with a prior systematic review by Cademartori et al, 41 finding no depression–periodontitis association, despite their meta-analysis indicating an independent relationship. The relationship between periodontitis and systemic conditions has recently gained significant attention. Notably, Sanz et al 22 demonstrated that periodontal inflammation contributes to cardiovascular diseases through direct bacteremia and indirect inflammatory mediator pathways. Their comprehensive review established periodontitis as an important risk factor for cardiovascular outcomes that warrants clinical attention. Additionally, Ramseier et al 23 established that periodontal treatment improves cardiovascular disease outcomes, highlighting the clinical significance of maintaining periodontal health in older adults. These findings underscore the importance of understanding how psychological factors might influence periodontal health, potentially mediating broader systemic health consequences.

The underlying mechanisms remain unclear, while included studies consistently demonstrate links between psychological factors and poor oral health behaviors. Both behavioral and biological pathways have been proposed. 34 42 43 Over the past decade, strong associations have emerged between stress/depression and unhealthy behaviors like smoking, excessive alcohol use, poor diet, and sedentary lifestyles. 11 17 19 20 44 By worsening oral health-related quality of life, depression and stress may also negatively impact self-perceived oral health. Biologically, these factors have been associated with reduced salivary flow, increased oral inflammation, and immune system dysfunction, 6 45 46 which may precipitate periodontitis. Further research is needed to elucidate the behavioral and biological mechanisms linking psychological factors to increased periodontitis susceptibility in older populations.

Regarding depression–periodontitis, eight studies were included, with four suggesting a positive association, three no association, and one an unclear relationship. Our mixed results align with prior reviews. Positive findings support hypotheses that depressed patients have heightened proinflammatory cytokines and acute phase proteins, thereby increasing the risk of periodontal disease. 47 48 Poor oral health from periodontitis may also increase depression risk via embarrassment, social isolation, etc. 10 49 However, Arias-Bujanda et al 48 noted that despite low risk of bias, high heterogeneity in depression and periodontitis assessments may obscure relationships.

In the depression–periodontitis studies, inconsistencies in the instruments used to assess depression (three different validated tools) likely contributed to the unclear associations found. Additionally, while all participants were older adults, differences in periodontitis assessment via clinical exam versus radiographs may explain the discordant results. This methodological heterogeneity highlights the need for standardized approaches in future research to enable more robust comparisons across studies. The use of different depression screening tools with varying sensitivity and specificity in geriatric populations could significantly impact findings, as noted in previous studies examining oral health–mental health relationships. Another systematic review also found no clear depression–periodontitis association, citing differences in methodological quality. 50 The inclusion of longitudinal, case–control, and cross-sectional studies with representative samples provides relative strength in the current review.

For stress, depression, and periodontitis, included studies generally found positive associations between exposures and outcomes. However, one study showed a complex stress–periodontitis relationship but no link to depression, 38 unlike a prior review indicating negative stress–periodontitis associations. 51 Methodological factors may again account for these discrepancies. Overall, the included studies were of high methodological quality, supporting a potential positive association between psychological factors and periodontitis in older adults that warrants further investigation.

The single study meeting our inclusion criteria that examined cognitive impairment–periodontitis associations found positive relationships. 37 Proposed mechanisms involve oral bacteria entering the bloodstream, breaching the blood–brain barrier, and contributing to neuroinflammation and cognitive decline. 52 This promising area of research has expanded considerably in recent years, with multiple studies and reviews exploring this relationship. The systematic review and meta-analysis by Guo et al 53 analyzed 20 studies and found significant associations between periodontal disease parameters and the risk of cognitive impairment. Additional research by Usuga-Vacca et al 56 has further substantiated these relationships, proposing common inflammatory pathways. While our strict inclusion criteria resulted in only one study being included, the broader literature strongly suggests this remains an important area for geriatric oral health research.

Several limitations in the included studies should be acknowledged when interpreting our findings. Methodological issues in periodontitis assessment in older adults were noted across multiple studies. 33 37 Periodontitis was assessed using varying methods (clinical examination, radiographs) and parameters (pocket depth, clinical attachment loss, bone loss), making direct comparisons challenging. Additionally, most studies used panoramic radiographs for bone loss assessment, which may not capture the full extent of periodontal destruction. Different psychological assessment tools further complicate interpretation, as these instruments vary in their psychometric properties and clinical thresholds, particularly for geriatric populations with potential comorbidities.

Limitations of this review include the limited literature available focusing specifically on older adults and the few psychological outcomes examined. The predominance of cross-sectional data limits causal inference about periodontitis and psychological factor relationships. Confounding factors were also poorly described. However, strengths include the GRADE evaluation demonstrating the methodological quality, including mostly high-quality studies with representative samples and adjusted analyses, and considering exposure–outcome directionality. Our review also employed a comprehensive search strategy across multiple databases, strict inclusion criteria focusing specifically on geriatric populations, and systematic assessment of evidence quality using established tools like NOS and GRADE, enhancing the reliability of our findings despite the limited available literature. This systematic review provides insights into potential associations between psychological factors and periodontitis in older adults that warrant further investigation using rigorous longitudinal designs. Future research should prioritize longitudinal studies examining bidirectional relationships between psychological factors and periodontitis; standardized assessment protocols for both periodontal parameters and psychological constructs; and consideration of potential mediating factors such as oral hygiene behaviors, systemic inflammation, and healthcare utilization patterns. Additionally, intervention studies evaluating whether addressing psychological factors improves periodontal outcomes would provide valuable clinical guidance for integrated approaches to geriatric oral healthcare.

## Conclusion

This systematic review and GRADE analysis indicate significant associations between psychological factors and periodontitis in older adults. Our findings demonstrate remarkably consistent relationships between stress and periodontitis, while the evidence for depression–periodontitis links showed more variable results. Cognitive impairment, though represented by fewer studies meeting our inclusion criteria, showed promising associations with periodontal disease that align with the broader literature on neuroinflammation and oral health. Although most included studies were cross-sectional, the single longitudinal study revealed increased risks of periodontitis in individuals with depression at both 6-month and 1-year follow-ups, suggesting temporal relationships that warrant further investigation. These findings have important clinical implications, suggesting that psychological assessment should be integrated into periodontal care protocols for elderly patients.

More longitudinal research is needed to examine bidirectional relationships and determine causality between periodontitis and psychological factors. Additionally, intervention studies exploring whether addressing psychological factors improves periodontal outcomes would provide valuable clinical guidance. Future research should employ standardized assessment methodologies for periodontal and psychological parameters to strengthen the evidence base and facilitate more robust cross-study comparisons. Additionally, further studies focused on oral health needs of older populations are recommended, particularly in middle- and low-income countries where geriatric growth is rapid. This review provides insights into links between psychological factors and periodontitis in the elderly, which may help clinicians and policymakers consider psychological well-being when addressing oral health issues in this demographic. In conclusion, findings indicate mental health factors should be considered in prevention and treatment strategies for periodontal disease in older adults.
