# Supplementary material for: The Impact of Psychological Factors on Periodontitis in the Elderly: A Systematic Review
Source: Eur J Dent. 2025 Jul 7;20(1):1–12. doi: 10.1055/s-0045-1809438 (PMC12890418; doi:10.1055/s-0045-1809438)
Supplement: Supplementary file 1 — Supplementary Material [file 10-1055-s-0045-1809438-s2524089.pdf]

Supplementary Table S1 Excluded studies with reason

No.	Author	Year	Title	Reason for exclusion
1.	Tong et al[1]	2010	Factors affecting oral health status in an elderly military veteran population in New Zealand	Study on the oral health status of the elderly
2.	Solis et al[2]	2004	Association of periodontal disease to anxiety and depression symptoms, and psychosocial stress factors	Age group from 19 to 61
3.	Braimoh et al[3]	2019	Oral health-related quality of life and associated factors of elderly population in Port Harcourt, Nigeria	No measure for psychological factors
4.	Cho et al[4]	2021	Prevalence and risk factors of anxiety and depression among the community-dwelling elderly in Nay Pyi Taw Union Territory, Myanmar	Measures other than oral disease
5.	Spector et al[5]	2020	Psychological Stress: A Predisposing and Exacerbating Factor in Periodontitis	No measures involved
6.	Ohi et al[6]	2021	Oral health-related quality of life is associated with the prevalence and development of depressive symptoms in older Japanese individuals: The Ohasama Study	Not related to periodontitis
7.	Ng and Keung Leung[7]	2006	A community study on the relationship between stress, coping, affective dispositions, and periodontal attachment loss	Age group between 25 and 75
8.	Moss et al[57]	1996	Exploratory case–control analysis of psychosocial factors and adult periodontitis	Age group between 25 and 75
9.	Li et al[8]	2022	Periodontitis and cognitive impairment in older adults: The mediating role of mitochondrial dysfunction	No psychological measures have been evaluated
10.	Laforgia et al[9]	2015	Assessment of psychopathological traits in a group of patients with adult chronic periodontitis: study on 108 cases and analysis of *compliance* during and after periodontal treatment	Age group between 25 and 67
11.	Moriya et al[10]	2022	Relationship between periodontal status and intellectual function among community-dwelling elderly persons	Not related to psychological factors
12.	Mendes et al[11]	2010	Analysis of socio-demographic and systemic health factors and the normative conditions of oral health care in a population of the Brazilian elderly	Not related to periodontitis
13.	Vettore et al[12]	2003	The relationship of stress and anxiety with chronic periodontitis	Age group between 45 and 67
14.	Terauchi et al[13]	2013	Associations among depression, anxiety, and somatic symptoms in peri- and postmenopausal women	Age group between 40 and 64
15.	Bevilacqua et al[14]	2019	Constructional apraxia screening and oral health among hospitalized older adults: A cross-sectional study.	Oral health outcomes like edentulism and systemic oral diseases were mentioned
16.	Puturidze et al[15]	2018	Relationship between general health, oral health, and healthy lifestyle in elderly population.	Review article
17.	Alkan et al[16]	2015	Relationship between psychological factors and oral health status and behaviors.	Other oral health outcomes were measured
18.	Kim et al[17]	2022	Risk of dementia according to the severity of chronic periodontitis in Korea: a nationwide retrospective cohort study	A questionnaire-based and calculated risk of dementia
19.	Bell et al[18]	2012	Dental anxiety and oral health outcomes among rural older adults	Systematic review
20.	Konishi et al[19]	2010	Factors associated with self-assessed oral health in the Japanese independent elderly.	A questionnaire-based study with all the oral health issues

(*Continued*)

 © 2025. The Author(s).

Supplementary Table S1 (*Continued*)

No.	Author	Year	Title	Reason for exclusion
21.	Hijryana et al[20]	2022	Impact of periodontal disease on the quality of life of older people in Indonesia: a qualitative study	Quality of life and other oral health measures
22.	Sharma et al[21]	2021	Evaluation of cognitive impairment in type 2 diabetic patients with chronic periodontitis: a cross-sectional study	Comorbidity like type 2 diabetes is measured
23.	Sekiguchi et al[22]	2020	Association between high psychological distress and poor oral health-related quality of life (OHQoL) in Japanese community-dwelling people: the Nagasaki Islands Study.	Quality of life and oral health-related issues
24.	Laforgia et al[9]	2015	Assessment of psychopathologic traits in a group of patients with adult chronic periodontitis: study on 108 cases and analysis of compliance during and after periodontal treatment.	Age group 19–79
25.	Hozuri et al[23]	2018	“The relationship between depression and periodontal indices in the elderly in Amirkola”	Age group 29–75
26.	Ng and Keung Leung[7]	2006	“A community study on the relationship between stress, coping, affective dispositions and periodontal attachment loss”	Coping mechanism studied
27.	Kim and Nam[24]	2020	“Comparison of diagnosed depression and self-reported depression symptom as a risk factor of periodontitis: analysis of 2016–2018 Korean National Health and Nutrition Examination Survey Data	Self-reported study and age group 17–75
28.	Gupta[25]	2014	“Periodontal disease,” caring for our aging population	Review article
29.	Ohi et al[6]	2022	“Oral health-related quality of life is associated with the prevalence and development of depressive symptoms in older Japanese individuals: The Ohasama Study”	Oral health-related quality of life
30.	Skośkiewicz-Malinowska et al[26]	2022	Oral health condition and occurrence of depression in the elderly”	All the oral health conditions
31.	Hybels et al[27]	2016	Trajectories of depressive symptoms and oral health outcomes in a community sample of older adults	Questionnaire-based study
32.	Salazar[28]	2013	The role of stress in periodontal disease progression in older adults”	Post doc thesis
33.	Sepolia and Verma[29]	2020	Periodontal oral health in geriatric patients: An observational study”	Observed periodontal status
34.	Subramanian et al[30]	2020	“Oxidative stress and oral diseases”	Age group 17–99
35.	Saletu et al[31]	2005	Controlled clinical and psychometric studies on the relation between periodontitis and depressive mood.	Age group 25–75

 © 2025. The Author(s).

Supplementary Table S2 PRISMA flowchart

Section and topic	Item #	Checklist item	Location where the item is reported
**Title**			
Title	1	Identify the report as a systematic review.	1
**Abstract**			
Abstract	2	See the PRISMA 2020 for abstracts checklist.	1
**Introduction**			
Rationale	3	Describe the rationale for the review in the context of existing knowledge.	2
Objectives	4	Provide an explicit statement of the objective(s) or question(s) the review addresses.	2–4
**Methods**			
Eligibility criteria	5	Specify the inclusion and exclusion criteria for the review and how studies were grouped for the syntheses.	4–5
Information sources	6	Specify all databases, registers, Web sites, organizations, reference lists and other sources searched or consulted to identify studies. Specify the date when each source was last searched or consulted.	5–6
Search strategy	7	Present the full search strategies for all databases, registers and Web sites, including any filters and limits used.	5–7
Selection process	8	Specify the methods used to decide whether a study met the inclusion criteria of the review, including how many reviewers screened each record and each report retrieved, whether they worked independently, and if applicable, details of automation tools used in the process.	7
Data collection process	9	Specify the methods used to collect data from reports, including how many reviewers collected data from each report, whether they worked independently, any processes for obtaining or confirming data from study investigators, and if applicable, details of automation tools used in the process.	7
Data items	10a	List and define all outcomes for which data were sought. Specify whether all results that were compatible with each outcome domain in each study were sought (e.g., for all measures, time points, analyses), and if not, the methods used to decide which results to collect.	8
	10b	List and define all other variables for which data were sought (e.g., participant and intervention characteristics, funding sources). Describe any assumptions made about any missing or unclear information.	8
Study risk of bias assessment	11	Specify the methods used to assess risk of bias in the included studies, including details of the tool(s) used, how many reviewers assessed each study and whether they worked independently, and if applicable, details of automation tools used in the process.	7
Effect measures	12	Specify for each outcome the effect measure(s) (e.g., risk ratio, mean difference) used in the synthesis or presentation of results.	7

(*Continued*)

 © 2025. The Author(s).

Supplementary Table S2 (*Continued*)

Section and topic	Item #	Checklist item	Location where the item is reported
Synthesis methods	13a	Describe the processes used to decide which studies were eligible for each synthesis (e.g., tabulating the study intervention characteristics and comparing against the planned groups for each synthesis (item #5)).	9
	13b	Describe any methods required to prepare the data for presentation or synthesis, such as handling of missing summary statistics, or data conversions.	10
	13c	Describe any methods used to tabulate or visually display the results of individual studies and syntheses.	11
	13d	Describe any methods used to synthesize results and provide a rationale for the choice(s). If meta-analysis was performed, describe the model (s), method(s) to identify the presence and extent of statistical heterogeneity, and software package (s) used.	5
	13e	Describe any methods used to explore possible causes of heterogeneity among study results (e.g., subgroup analysis, meta-regression).	5
	13f	Describe any sensitivity analyses conducted to assess the robustness of the synthesized results.	7
Reporting bias assessment	14	Describe any methods used to assess risk of bias due to missing results in a synthesis (arising from reporting biases).	6
Certainty assessment	15	Describe any methods used to assess certainty (or confidence) in the body of evidence for an outcome.	7
**Results**			
Study selection	16a	Describe the results of the search and selection process, from the number of records identified in the search to the number of studies included in the review, ideally using a flow diagram.	9
	16b	Cite studies that might appear to meet the inclusion criteria, but which were excluded, and explain why they were excluded.	9
Study characteristics	17	Cite each included study and present its characteristics.	10
Risk of bias in studies	18	Present assessments of risk of bias for each included study.	13–15
Results of individual studies	19	For all outcomes, present, for each study: (a) summary statistics for each group (where appropriate) and (b) an effect estimate and its precision (e.g., confidence/credible interval), ideally using structured tables or plots.	10–13
Results of syntheses	20a	For each synthesis, briefly summarize the characteristics and risk of bias among contributing studies.	10
	20b	Present results of all statistical syntheses conducted. If meta-analysis was done, present for each the summary estimate and its precision (e.g., confidence/credible interval) and measures of statistical heterogeneity. If comparing groups, describe the direction of the effect.	12

 © 2025. The Author(s).

Supplementary Table S2 (*Continued*)

Section and topic	Item #	Checklist item	Location where the item is reported
	20c	Present results of all investigations of possible causes of heterogeneity among study results.	13
	20d	Present results of all sensitivity analyses conducted to assess the robustness of the synthesized results.	11
Reporting biases	21	Present assessments of risk of bias due to missing results (arising from reporting biases) for each synthesis assessed.	15
Certainty of evidence	22	Present assessments of certainty (or confidence) in the body of evidence for each outcome assessed.	16
**Discussion**			
Discussion	23a	Provide a general interpretation of the results in the context of other evidence.	18–20
	23b	Discuss any limitations of the evidence included in the review.	19
	23c	Discuss any limitations of the review processes used.	20
	23d	Discuss implications of the results for practice, policy, and future research.	20
**Other information**			
Registration and protocol	24a	Provide registration information for the review, including the register name and registration number, or state that the review was not registered.	4
	24b	Indicate where the review protocol can be accessed, or state that a protocol was not prepared.	
	24c	Describe and explain any amendments to information provided at registration or in the protocol.	
Support	25	Describe sources of financial or non-financial support for the review, and the role of the funders or sponsors in the review.	
Competing interests	26	Declare any competing interests of review authors.	
Availability of data, code and other materials	27	Report which of the following are publicly available and where they can be found: template data collection forms; data extracted from included studies; data used for all analyses; analytic code; any other materials used in the review.	

 © 2025. The Author(s).

References

1 Tong D, Dawson J, Love R. Factors affecting oral health status in an elderly military veteran population in New Zealand. J Mil Veterans Health 2010;18(03):12–17

2 Solis ACO, Lotufo RFM, Pannuti CM, Brunheiro EC, Marques AH, Lotufo-Neto F. Association of periodontal disease to anxiety and depression symptoms, and psychosocial stress factors. J Clin Periodontol 2004;31(08):633–638

3 Braimoh OB, Alade GO. Oral health-related quality of life and associated factors of elderly population in Port Harcourt, Nigeria. Saudi Journal of Oral Sciences 2019;6(01):18–24

4 Cho SM, Saw YM, Saw TN, et al. Prevalence and risk factors of anxiety and depression among the community-dwelling elderly in Nay Pyi Taw Union Territory, Myanmar. Sci Rep 2021; 11(01):9763

5 Spector AM, Postolache TT, Akram F, Scott AJ, Wadhawan A, Reynolds MA. Psychological stress: a predisposing and exacerbating factor in periodontitis. Curr Oral Health Rep 2020; 7:208–215

6 Ohi T, Murakami T, Komiyama T, et al. Oral health-related quality of life is associated with the prevalence and development of depressive symptoms in older Japanese individuals: The Ohasama Study. Gerodontology 2022;39(02):204–212

7 Ng SK, Keung Leung W. A community study on the relationship between stress, coping, affective dispositions and periodontal attachment loss. Community Dent Oral Epidemiol 2006;34(04): 252–266

8 Li A, Du M, Chen Y, et al. Periodontitis and cognitive impairment in older adults: The mediating role of mitochondrial dysfunction. J Periodontol 2022;93(09):1302–1313

9 Laforgia A, Corsalini M, Stefanachi G, Pettini F, Di Venere D. Assessment of psychopathologic traits in a group of patients with adult chronic periodontitis: study on 108 cases and analysis of compliance during and after periodontal treatment. Int J Med Sci 2015;12(10):832–839

10 Moriya S, Tei K, Toyoshita Y, Koshino H, Inoue N, Miura H. Relationship between periodontal status and intellectual function among community-dwelling elderly persons. Gerodontology 2012;29(02):e368–e374

11 Mendes DC, Poswar FdeO, de Oliveira MVM, et al. Analysis of socio-demographic and systemic health factors and the normative conditions of oral health care in a population of the Brazilian elderly. Gerodontology 2012;29(02):e206–e214

12 Vettore MV, Leão ATT, Monteiro Da Silva AM, Quintanilha RS, Lamarca GA. The relationship of stress and anxiety with chronic periodontitis. J Clin Periodontol 2003;30(05):394–402

13 Terauchi M, Hiramitsu S, Akiyoshi M, et al. Associations among depression, anxiety and somatic symptoms in peri- and postmenopausal women. J Obstet Gynaecol Res 2013;39(05): 1007–1013

14 Bevilacqua L, Severin A, Russi E, et al. Constructional apraxia screening and oral health among hospitalized older adults: a cross-sectional study. Spec Care Dentist 2019;39(05):491–496

15 Puturidze S, Margvelashvili M, Bilder L, Kalandadze M, Margvelashvili V. Relationship between general health, oral health and healthy lifestyle in elderly population (Review). Georgian Med News 2018;1(Issue):17–21

16 Alkan A, Cakmak O, Yilmaz S, Cebi T, Gurgan C. Relationship between psychological factors and oral health status and behaviours. Oral Health Prev Dent 2015;13(04):331–339

17 Kim SR, Son M, Kim YR, Kang HK. Risk of dementia according to the severity of chronic periodontitis in Korea: a nationwide retrospective cohort study. Epidemiol Health 2022;44:e2022077

18 Bell RA, Arcury TA, Anderson AM, et al. Dental anxiety and oral health outcomes among rural older adults. J Public Health Dent 2012;72(01):53–59

19 Konishi C, Hakuta C, Ueno M, Shinada K, Wright FA, Kawaguchi Y. Factors associated with self-assessed oral health in the Japanese independent elderly. Gerodontology 2010;27(01):53–61

20 Hijryana M, MacDougall M, Ariani N, Kusdhany LS, Walls AWG. Impact of periodontal disease on the quality of life of older people in Indonesia: a qualitative study. JDR Clin Trans Res 2022;7(04): 360–370

21 Sharma S, Nayak SU, Uppoor A, Rao S, Pai K, Natarajan S. Evaluation of cognitive impairment in type 2 diabetic patients with chronic periodontitis: a cross-sectional study. J Int Soc Prev Community Dent 2021;11(01):50–57

22 Sekiguchi A, Kawashiri SY, Hayashida H, et al. Association between high psychological distress and poor oral health-related quality of life (OHQoL) in Japanese community-dwelling people: The Nagasaki Islands Study. Environ Health Prev Med 2020;25(01):82

23 Hozuri M, Khirkhah F, Hossieni SR, et al. The relationship between depression and periodontal indices in the elderly in Amirkola. J Babol Univ Med Sci 2020;22:143–149

24 Kim SR, Nam SH. Comparison of diagnosed depression and self-reported depression symptom as a risk factor of periodontitis: analysis of 2016-2018 Korean National Health and Nutrition Examination Survey Data. Int J Environ Res Public Health 2021; 18(03):871

25 Gupta S. Periodontal disease. In: Friedman PK. eds. Geriatric Dentistry:Caring for Our Aging Population. Wiley-Blackwell; 2014:107

26 Skośkiewicz-Malinowska K, Malicka B, Ziętek M, Kaczmarek U. Oral health condition and occurrence of depression in the elderly. Medicine (Baltimore) 2018;97(41):e12490

27 Hybels CF, Bennett JM, Landerman LR, Liang J, Plassman BL, Wu B. Trajectories of depressive symptoms and oral health outcomes in a community sample of older adults. Int J Geriatr Psychiatry 2016; 31(01):83–91

28 Salazar CR. The role of stress in periodontal disease progression in older adults. Postdoc J 2013;1(11):15–26

29 Sepolia S, Verma P. Periodontal oral health in geriatric patients: an observational study. J Adv Med Dent Scie Res 2020;8(11):223–228

30 Subramanian AK, Narayan V, Navaneethan R. Oxidative Stress and Oral Diseases. In: Maurya PK, Dua K. eds. Role of Oxidative Stress in Pathophysiology of Diseases. Singapore: Springer; 2020:1–12

31 Saletu A, Pirker-Frühauf H, Saletu F, Linzmayer L, Anderer P, Matejka M. Controlled clinical and psychometric studies on the relation between periodontitis and depressive mood. J Clin Periodontol 2005;32(12):1219–1225

 © 2025. The Author(s).
